# Glutathione Peroxidase 4 as a Therapeutic Target for Anti-Colorectal Cancer Drug-Tolerant Persister Cells

**DOI:** 10.3389/fonc.2022.913669

**Published:** 2022-06-03

**Authors:** Xiaoli Zhang, Yiming Ma, Jianhui Ma, Lan Yang, Qingzhi Song, Hongying Wang, Guoqing Lv

**Affiliations:** ^1^ Department of Gastrointestinal Surgery, Peking University Shenzhen Hospital, Shenzhen Peking University-The Hong Kong University of Science and Technology Medical Center, Shenzhen, China; ^2^ State Key Laboratory of Explosion Science and Technology, Beijing Institute of Technology, Beijing, China; ^3^ State Key Laboratory of Molecular Oncology, National Cancer Center/National Clinical Research Center for Cancer/Cancer Hospital, Chinese Academy of Medical Sciences and Peking Union Medical College, Beijing, China; ^4^ Department of Gastroenterology of Shenzhen Second People’s Hospital, The First Affiliated Hospital of Shenzhen University, Health Science Center, Shenzhen Second People’s Hospital, Shenzhen, China

**Keywords:** colorectal cancer, drug tolerance, ferroptosis, glutathione peroxidase 4 (GPX4), ferrous iron

## Abstract

**Background:**

Despite the effectiveness of chemotherapy and targeted therapy for colorectal cancer, drug resistance drives therapy failure and tumor relapse. Increasing evidence has suggested that cancer cells can enter a reversible drug-tolerant persister state to survive chemotherapy or targeted agents. However, the traits and treatable vulnerabilities of anti-colorectal cancer drug-tolerant persister cells is not yet known.

**Methods:**

In this study, we established 5-fluorouracil and AZ628-tolerant persister cell models in two colorectal cancer cell lines, namely HCT116 and SW620, and revealed the characteristics of colorectal cancer persister cells by cell viability assay and flow cytometry. We investigated the efficacy and mechanism of ferroptosis inducers RSL3 and FIN56 on persister cells, which are glutathione peroxidase 4 inhibitors. In the xenograft mouse model, we further evaluated the inhibitory effect of RSL3 on tumor regrowth.

**Results:**

Colorectal cancer persister cells, which were enriched in the residual cancer cell population, exhibited reduced drug sensitivity, were largely quiescent and expressed high levels of stem cell-related genes and mesenchymal markers but not epithelial markers. The persister cells were more sensitive and underwent ferroptosis induced by glutathione peroxidase 4 inhibitors. Mechanistically, glutathione peroxidase 4 and ferrous iron, which are pivotal ferroptosis regulators, were upregulated in residual cells or tumors, and were hence potential therapeutic targets of persister cells. In the xenograft model, we confirmed that inhibition of glutathione peroxidase 4 restrained tumor regrowth after discontinuation of anti-cancer drug treatment. Moreover, biopsies obtained from patients with colorectal cancer undergoing neoadjuvant chemoradiotherapy revealed upregulated glutathione peroxidase 4 and ferritin heavy chain 1. High glutathione peroxidase 4 expression correlates with a worse prognosis in colorectal cancer patients.

**Conclusions:**

Our work reveals that the upregulated glutathione peroxidase 4 and ferrous iron in anti-colorectal cancer drug-tolerant persister cells were potential therapeutic targets. Glutathione peroxidase 4 inhibition combined with chemotherapy or targeted therapy may be a promising therapy for colorectal cancer.

## Introduction

Colorectal cancer (CRC) is the third most frequent cancer, with nearly 1.89 million new cases diagnosed in 2020, and the second most common cause of cancer-related death (1).Surgery is the most effective treatment for CRC, especially during the early tumor stages. For CRC patients with distant metastasis, neoadjuvant or adjuvant treatments can be introduced, composed of chemotherapy or targeted therapy (2). The first-line chemotherapeutic drug for CRC treatment is 5-fluorouracil (5-FU), which is a synthetic fluorinated pyrimidine analog that requires intracellular conversion into its active metabolites (3). AZ628, a pan-RAF kinase inhibitor, has been shown to exhibit excellent efficacy in cells harboring the BRAF or KRAS mutations that occur in ~10% and 40% of CRC cases, respectively (4–6), along with promising preclinical results (7). Patients with CRC usually undergo multiple chemotherapy cycles throughout their courses (8). Upon treatment removal, often referred to as a “drug holiday”, tumor cells can regrow or relapse. Moreover, drug resistance limits durable clinical benefits and the survival outcomes of CRC remain suboptimal (9).

Within the same patient or even the same tumor, multiple mechanisms of drug resistance can coexist, and this is generally thought to be due to rare, stochastic genetic alterations (10). However, more recent findings have revealed a non-mutational drug-tolerant persister (DTP) state (11). Key features of DTP cells include their quiescence and their ability to remain viable under conditions where the vast majority of the cell population is rapidly killed (11, 12). Furthermore, DTP cells represent a reversible state where, upon removal of the drug, they resume proliferation and growth and remain sensitive to the initial therapy (13, 14). Persister cells can be generated *in vitro* from commercial cell lines or *in vivo* from patient-derived xenografts, and they present across various kinds of tumors (11, 14). However, the characteristics of DTPs derived from CRC cells treated with the strong selective pressure of high concentrations of chemotherapy drug 5-FU or the targeted agent AZ628 remain largely unknown.

Drug-tolerant persister cells may eventually acquire irreversible resistance-conferring genetic mutations and *bona fide* drug-resistance mechanisms to avoid eradication by prolonged lethal exposures (10). The DTP state provides a latent reservoir of cells for the emergence of drug resistance. Pharmacological disruption of this “intermediate” state, therefore, presents a therapeutic opportunity to prevent drug resistance and impede tumor relapse (15). Ferroptosis, which is a type of cell death dependent on iron, reactive oxygen species (ROS), and lipid peroxidation, has been reported as a promising therapeutic strategy to selectively deplete DTP cells in breast, melanoma and ovarian cancer (16, 17). Iron accumulation, particularly ferrous iron (Fe^2+^), can create ROS by participating in the Fenton reaction, thereby triggering ferroptosis (18). Additionally, the lipid ROS scavenger gutathione peroxidase 4 (GPX4) can convert lipid peroxides into non-toxic lipid alcohols to protect cells against ferroptotic death (19–21). Drug-tolerant persister breast cancer cells are vulnerable to ferroptosis induced by GPX4 inhibition (16). However, the vulnerability of CRC DTPs derived from chemotherapy or targeted agents to ferroptosis and the underling mechanisms remain unclear.

In this study, we followed a previously established procedure (16) and established DTP models based on CRC cells exposed to lethal concentrations of 5-FU or AZ628. We revealed the features of CRC DTP cells and found these cells were sensitive to GPX4 inhibitors-induced ferroptosis *in vitro*. In the xenograft model, we confirmed inhibition of tumor regrowth by 1S,3R-RSL3 (RSL3) after discontinuation of 5-FU treatment. Mechanistic studies showed that GPX4 expression and ferrous iron were increased in 5-FU and AZ628-derived CRC persister cells, residual subcutaneous transplantation tumors, and colorectal cancer patients undergoing neoadjuvant chemoradiotherapy. The upregulated GPX4 was identified as a likely target molecule in targeted therapy for CRC persister cells. Our study clarifies the nature of CRC DTPs in detail, and provides a potential therapeutic strategy to eradicate them, prevent subsequent drug resistance, and delay tumor relapse.

## Materials and Methods

### Chemicals and Reagents

5-Fluorouracil (Cat# F6627), erastin (Cat# E7781) and ferrostatin-1 (Cat# SML0583) were purchased from Sigma-Aldrich (St. Louis, MO, USA). AZ628 (Cat# S2746), (1S, 3R)-RSL3 (Cat# S8155) and FIN56 (Cat# S8254) were purchased from Selleck Chemicals (Houston, TX, USA). BODIPY™ 581/591C11 (Cat# D3861) was obtained from Thermo Fisher Scientific (Waltham, MA, USA). FeRhoNox-1 (an Fe^2+^ indicator, Cat# MX4558) was purchased from MKBio (Shanghai, China).

### Cell Culture

The human colon cancer cell lines HCT116 and SW620, purchased from American Type Culture Collection (Manassas, VA, USA), were cultured in RPMI medium and DMEM/F12 (HyClone, Logan, UT, USA) respectively, supplemented with 10% foetal bovine serum (HyClone) and 1% penicillin and streptomycin. The cells were cultured at 37°C in a humidified, 5% CO_2_ environment.

### Persister Cell Derivation and Treatment

Persister cells were derived from colorectal cancer cell lines HCT116 (KRAS^G13D^) and SW620 (KRAS^G12V^) treated with 5-FU and AZ628, for at least 9 days with fresh drug added every 3 days, and then stained with crystal violet or counted using a Countess Automated Cell Counter (Invitrogen, Carlsbad, CA, USA). Pre-derived persister cells from 5-FU or AZ628 treatment were allowed to regrow upon removal of the drug. To testing ferroptosis inducers treatment, parental cells, persister cells, and regrown cells were treated with inducers alone or in combination with ferrostatin-1 (Fer-1). Cell viability was assessed using the MTT assay.

### Reverse Transcription-Quantitative Polymerase Chain Reaction

The total RNA content was isolated from cells or the tumor using TRIzol™ reagent (Invitrogen), treated with DNase I (Thermo Fisher Scientific, Cat# EN0521), and then reverse transcribed into cDNA using a Maximal First Strand cDNA Synthesis Kit (Thermo Fisher Scientific, Cat# K1622). Real-time PCR was performed using an S1000 PCR instrument (Bio-Rad, Hercules, CA, USA) to quantify gene expression. The PCR data were normalized to glyceraldehyde-3-phosphate dehydrogenase expression at the mRNA level. The primers used are listed in **Supplementary Table S1**.

### Western Blot

Total protein content of cell lines or the tumor was extracted by lysis in radioimmunoprecipitation assay buffer containing protease inhibitor cocktail and the protein phosphatase inhibitor, and the total protein concentration was quantified by the BCA (bicinchoninic acid) method. Equal amounts of protein lysates were resolved by sodium dodecyl sulfate -polyacrylamide gel electrophoresis and were transferred to polyvinylidene difluoride membranes. The membranes were blocked at 37°C for 1.5 h in 5% milk and then incubated 12-16 h at 4°C with the following primary antibodies: COX2 (Abcam, Cat# ab179800), AKR1C1 (Abcam, Cat# ab192785), FTH1 (Abcam, Cat# ab75973), GPX4 (Abcam, Cat# ab125066) and β-actin (Invitrogen, Cat# A1978). The membranes were then incubated with horseradish peroxidase-linked secondary antibodies (Cell Signaling Technology) and the signals were visualized by enhanced chemiluminescence using an Amersham Imager 600 (GE Healthcare). The intensities of the signals were quantified using the ImageJ software.

### Immunofluorescence Staining

Immunofluorescence staining was performed as described (22). Briefly, the cells were fixed in 4% formaldehyde and then incubated with 5% bovine serum albumin. The primary antibodies used were anti-GPX4 antibody (Proteintech, Cat# 67763, 1:100), and anti-FTH1 antibody (Abcam, Cat# ab75973, 1:200).

### Lipid Peroxidation Staining

Cells were incubated for 30 min at 37°C with BODIPY™ 581/591C11 to detect lipid peroxidation. After incubation, the cells were harvested by trypsinization. The level of lipid peroxidation was determined by imaging of the stained cells using an UltraVIEWVoX double spinning disk confocal microscope (PerkinElmer Life Sciences) or by flow cytometry analysis.

### Ferrous Iron Staining

Cells were incubated for 1 h at 37°C with FeRhoNox-1 (an Fe^2+^ indicator) to detect ferrous iron. The cells were then harvested by trypsinization, and the level of ferrous iron was determined by imaging using a confocal microscope or by flow cytometry analysis.

### Measurement of Cell Cycle Progression

Cell cycle progression was measured by flow cytometry as previously described (23). Briefly, adherent cells were harvested and washed twice with phosphate-buffered saline. The cells were fixed overnight in 70% ethanol at -20°C. The cells were then stained with 1 mg/mL propidium iodide (containing 1 mg/mL of RNase A) for 30 min in the dark at room temperature and then analyzed using a flow cytometer (BD LSR II). The percentages of cells in the indicated cell cycle phases were analyzed using FlowJo software.

### Mouse Xenograft Studies

All protocols for animal procedures were approved by the Animal Care and Use Committee of Peking University Shenzhen Hospital (No. 2022206). Six-to-eight-week-old male nude mice were subcutaneously injected with 1 × 10^6^ HCT116 cells in 100 μL. The mice were observed daily for tumor growth. When the average tumor volume reached approximately 100 mm^3^, the mice were divided randomly into four groups (n = 5). We administered saline plus vehicle (control) and RSL3 or 5-FU alone and in combination (i.e., 5-FU plus RSL3). The drug 5-FU was administrated daily at a dose of 30 mg/kg intraperitoneally, while normal saline was used as a control. RSL3 was injected intraperitoneally at 50 mg/kg dosage every 3 days. To determine the effect of each therapeutic regimen, the tumor volumes were measured once every 2 days until 20 days from therapy initiation. The lengths (L) and widths (W) of the tumors were measured, and the tumor volumes were calculated as (L × W^2^)/2.

### Human Clinical Samples

The use of human tissues was approved by the Institutional Review Board of Peking University Shenzhen Hospital (No. 2021771). Six colorectal cancer tissues were obtained, along with informed consent, from the CRC patients. For immunohistochemistry staining, cancer tissues were dissected and fixed in 10% neutral buffered formalin. Three patients received chemoradiotherapy before surgical resection, while three did not.

### Statistical Analysis

All experiments were repeated independently at least three times unless stated otherwise. Statistical analyses were performed using GraphPad Prism software (version 8.0). Data are presented as means ± Standard Deviation. Comparisons between two groups were made using Student’s t-test for unpaired data. Comparisons of more than two groups were performed using analysis of variance (ANOVA) with Tukey’s test. For all tests, *p* values less than 0.05 were considered statistically significant (*p < 0.05, **p < 0.01, and ***p < 0.001).

## Results

### Establishment of CRC Drug-Tolerant Persister Cell Models

**Figure 1:**
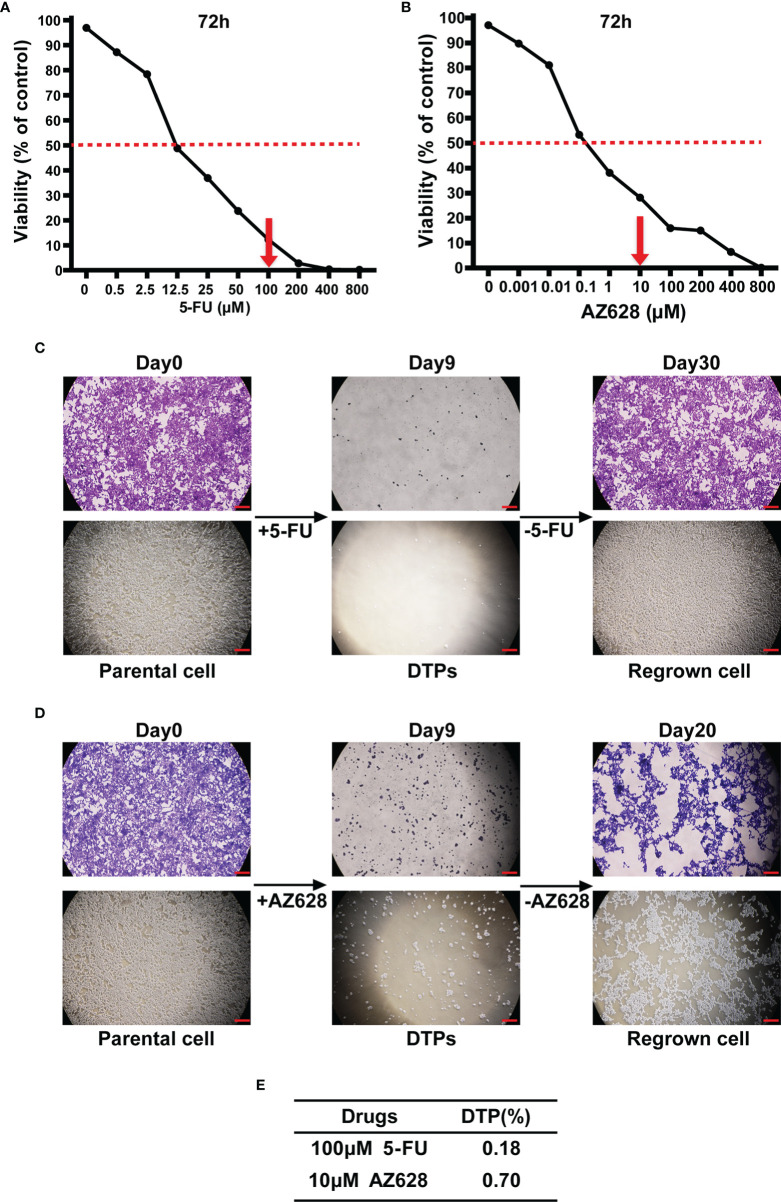
Establishment of CRC drug-tolerant persister cell models. **(A, B)** Survival curves representing the viability of HCT116 cells treated with the indicated concentrations of 5-fluorouracil (5-FU) or AZ628 for 72 hours. Each data point is expressed as the percentage of surviving cells relative to untreated controls. The dashed line corresponds to 50% cell killing. The grey arrow indicates the concentration of 5-FU (100 μM) or AZ628 (10 μM) used to generate DTP cells. **(C, D)** Representative pictures of crystal violet staining of HCT116 cells that were either untreated (left) or treated with 100 μM 5-FU or 10 μM AZ628 for 9 days (middle, fresh drug was added every 3 days) or removal of drugs (right). At the indicated times, representative microscope images were taken (lower panel, scale: 100 μm). **(E)** Following 9 days of treatment, surviving DTP cells were quantified (ratio to Day0 cell counts). Data are presented as means *±* SD of three independent experiments.

While modeling the response to the chemotherapeutic drug 5-FU in the CRC cell line HCT116, which has exquisite sensitivity, we observed that the vast majority of the cells were killed within 9 days of exposure to the drug at a concentration 10-fold higher than the IC_50_ value (**Figure 1A**). Approximately 0.18% of the starting population survived and settled into a persister state characterized by drug tolerance and negligible growth (**Figures 1C, E** **)**. Drug removal for 21 days allowed the persister cells to regrow (**Figure 1C**). Similarly, HCT116 cells treated with the pan-Raf kinase inhibitor AZ628 (~100-fold higher than the IC_50_) also yielded a small percentage of DTPs, amounting to ~0.70% of the starting population (**Figures 1B, D, E** **)**. In addition, another CRC cell line SW620 drug-tolerant persister cell models had been established as well (**Supplementary Figure S1**).

### Characteristics of The Drug-Tolerant CRC Persister Cells

**Figure 2:**
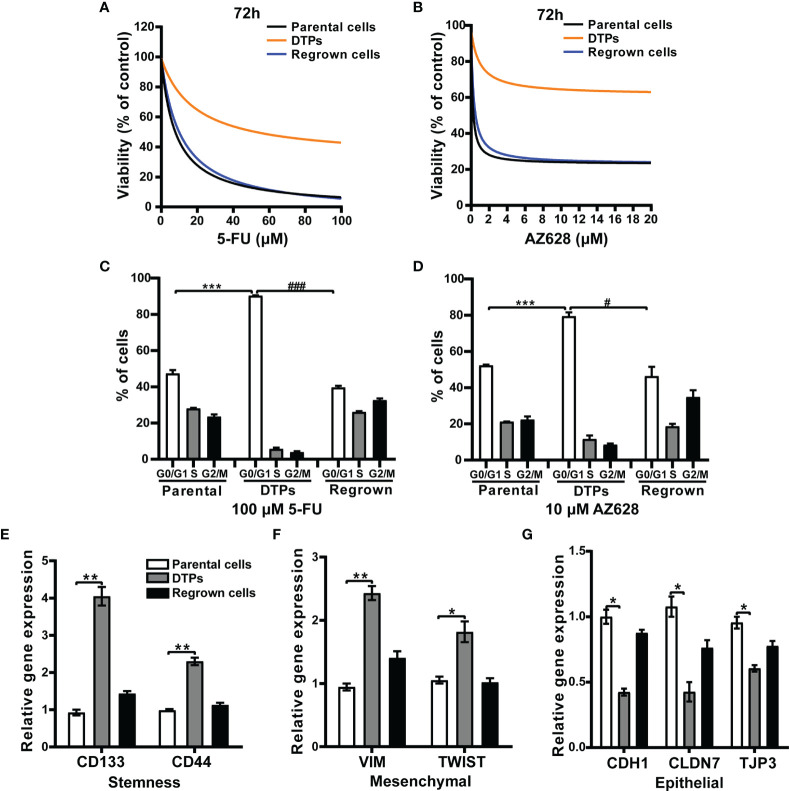
The characteristics of drug-tolerant CRC persister cells. **(A)** Survival curves representing the viability of HCT116 parental cells, DTP cells generated by selection in 100 μM 5-FU, and regrown cells upon treatment with the indicated 5-FU concentrations for 72 hours. Each data point is expressed as the percentage of surviving cells relative to untreated controls. **(B)** Similar results were observed with AZ628. **(C, D)** Cells were analyzed by FACS to determine the percentage of cells in the indicated cell cycle phases. **(E-G)** The relative mRNA levels of **(E)** stemness (*CD133* and *CD44*), **(F)** mesenchymal (*VIM* and *TWIST*), and **(G)** epithelial (*CDH1 CLDN7 TJP3*) markers measured by real-time PCR for the three different conditions under 5-FU treatment. Data are presented as means ± SD of two independent experiments. Statistical significance was determined using one-way ANOVA with Tukey’s test. *p < 0.05 vs. parental group; *^#^p <* 0.05 vs. DTP group, **p < 0.01, and ***p < 0.001 vs. parental group; *^###^p <* 0.001 vs. DTP group.

Similar to previous reports regarding non-small cell lung cancer persister cells (11), the CRC DTP cells exhibited reduced sensitivity to the modeling drugs. CRC persister cells propagated in drug-free media resumed growth and rapidly reacquired sensitivity to 5-FU or AZ628 (**Figures 2A, B** **)**. Furthermore, flow cytometry analysis showed that CRC DTP cells had a greater proportion of cells in G0/G1 phase (5-FU DTPs 90.3 ± 0.6%; AZ628 DTPs 79.5 ± 1.5%) compared to parental cells (50.4 ± 4.2%) (**Figures 2C, D** **)**. The residual cells were arrested at the G0/G1 phase of the cell cycle, and the percentage of cells in the S phase was reduced, while the parental and regrown cells showed the similar cell cycle distribution. This is probably the reason why the persister cells were quiescent. Considering the reported links between drug resistance and a “cancer stem cell” phenotype, we examined cancer stem cell markers. We observed upregulation of the stemness markers *CD133* and *CD44* in the CRC DTP cells (**Figure 2E**). We also observed upregulation of mesenchymal markers (*VIM* and *TWIST*) and downregulation of epithelial markers (*CDH1*, *CLDN7* and *TJP3*, **Figures 2F, G** **)**, consistent with earlier observations in other persister cell models (16). These findings confirmed that the 5-FU and AZ628-tolerant CRC persister cell models had been successfully established.

### CRC Persister Cells Are Vulnerable to Ferroptosis

**Figure 3:**
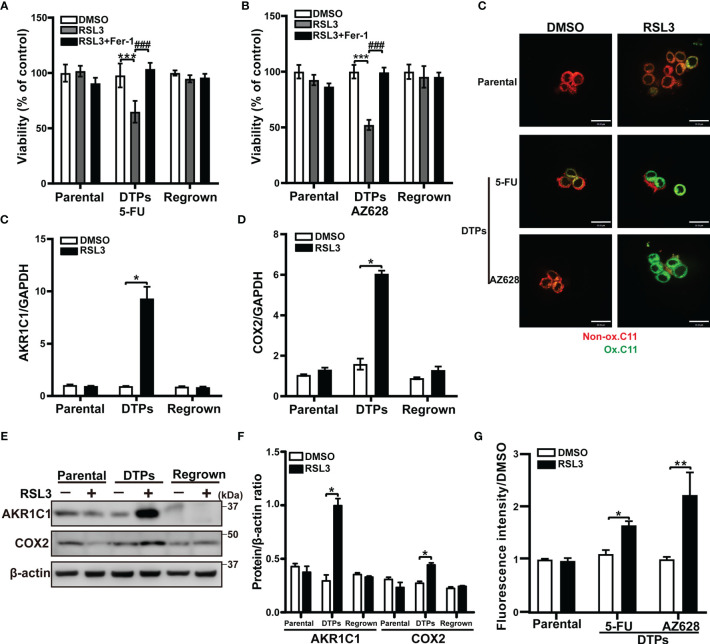
CRC persister cells are vulnerable to ferroptosis. **(A, B)** MTT assay of HCT116 parental and DTP cells generated by selection in **(A)** 100 μM 5-FU or **(B)** 10 μM AZ628 and regrown for 48 hours ± 1 µM 1S,3R-RSL3 (RSL3) and 2 µM ferrostatin-1 (Fer-1). **(C, D)** The relative mRNA levels of **(C)** *AKR1C1* and **(D)** *COX2* measured by real-time PCR in the three different conditions under 5-FU treatment with or without 1 µM RSL3. **(E, F) (E)** Western blot analysis of AKR1C1 and COX2 in the three different conditions under 5-FU treatment with or without 1 µM RSL3. **(F)** AKR1C1 and COX2 densitometry relative to β-actin. **(G, H)** Confocal microscopy **(G)** and flow cytometry **(H)** analysis of lipid peroxidation levels based on 5 μM BODIPY™ 581/591 C11 signal. Red and green fluorescence indicates non-oxidized (Non-ox.) and oxidized lipids (Ox.), respectively. Data are presented as means ± SD of three independent experiments. Statistical significance was determined using one-way ANOVA with Tukey’s test. *p < 0.05, **p < 0.01, and ***p < 0.001 vs. DMSO group; *^###^p <* 0.001 vs. RSL only group.

To effectively eliminate CRC DTP cells, we sought to identify therapeutic vulnerabilities in persister cells that are absent in parental and regrown cells. It has been reported that cells in a high mesenchymal or stem state are selectively sensitive to ferroptosis, which is a regulated cell death executed by lipid hydroperoxides (18). We found that the ferroptosis inducers RSL3 and FIN56 (GPX4 inhibitors) were both selectively lethal to CRC persister cells, while its effects on parental and regrown cells were minimal, as was another ferroptosis inducer erastin. Cell death induced by these inducers was completely reversed by the small-molecule inhibitor of ferroptosis Fer-1 (**Figures 3A, B** **and** **Supplementary Figures S2A–C**). In keeping with this, the mRNA and protein levels of the ferroptosis biomarkers AKR1C1 and COX2 were upregulated in RSL3-treated DTPs, but not in the parental or regrown cells (**Figures 3C–F**, and **Supplementary Figures S2D, E**). RSL3 increased oxidized membrane lipid levels, as measured by confocal and flow cytometry using the dye C11-BODIPY in CRC DTP cells compared to the parental cells, supporting the notion of increased susceptibility of CRC persister cells to ferroptosis after 5-FU or AZ628 treatment (**Figures 3G, H** **)**. These findings indicate that ferroptotic death is a promising strategy to selectively eradicate CRC persister cells that arise with diverse therapeutic regimens.

### Elevated GPX4 and Ferrous Iron Are Potential Therapeutic Targets of CRC Persister Cells and Tumors

**Figure 4:**
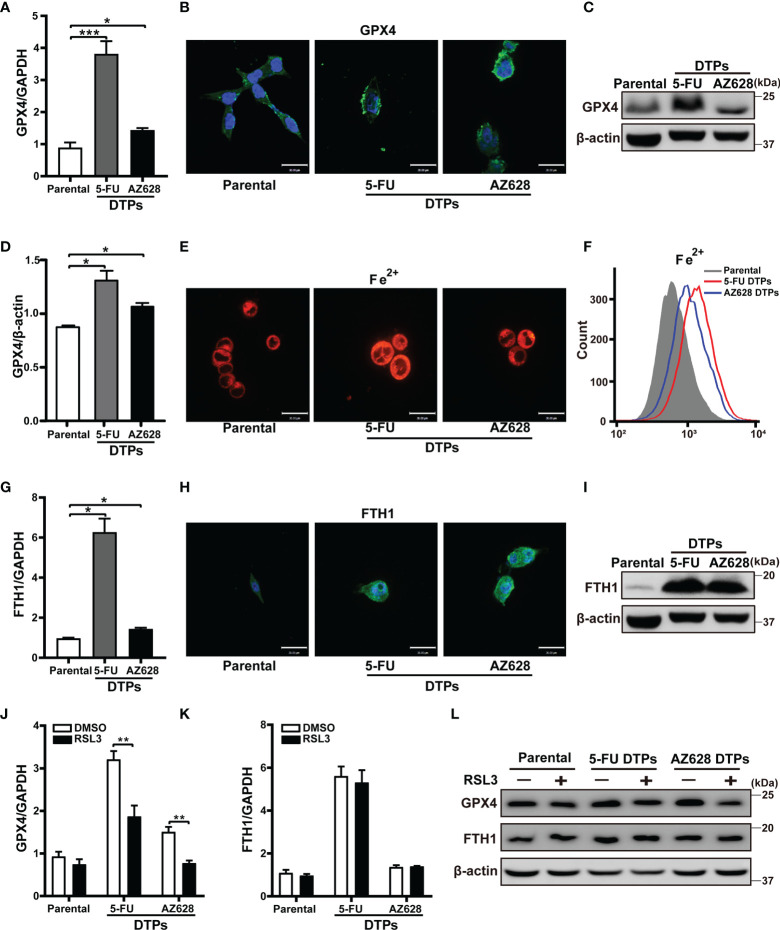
Elevated GPX4 and ferrous iron are potential therapeutic targets of CRC persister cells. **(A)** The *GPX4* mRNA levels were measured by real-time PCR in HCT116 parental and persister cells derived by 5-FU or AZ628 treatment. **(B)** Representative immunofluorescence images of GPX4 staining. Blue and green fluorescence indicate nuclear and GPX4, respectively (scale: 30 μm). **(C, D) (C)** Western blot analysis of GPX4, (D) GPX4 densitometry relative to β-actin. **(E, F) (E)** confocal microscopy and **(F)** flow cytometry analysis of the level of ferrous ions in the cytoplasm using 2 μM FeRhoNox-1 (scale: 30 μm). **(G)** The *FTH1* mRNA levels were measured by real-time PCR in HCT116 parental and persister cells derived by 5-FU or AZ628 treatment. **(H)** Representative immunofluorescence images of FTH1 staining. Blue and green fluorescence indicate nuclear and FTH1 staining, respectively (scale: 30 μm). **(I)** Western blot analysis of FTH1. **(J, K) (J)** *GPX4* and **(K)** *FTH1* mRNA levels were measured by real-time PCR in HCT116 parental and DTP cells for 48 hours with or without 1 µM RSL3. **(L)** Western blot analysis of GPX4 and FTH1. Data are presented as means ± SD of three independent experiments. Statistical significance was determined using one-way ANOVA with Tukey’s test. *p < 0.05, **p < 0.01, and ***p < 0.001.

**Figure 5:**
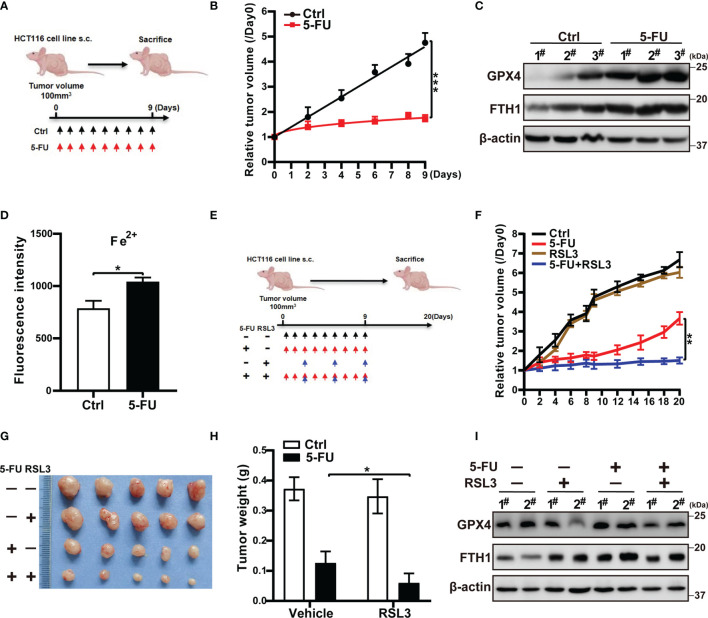
The ferroptosis inducer RSL3 suppresses CRC regrowth after 5-FU therapy cessation *in vivo*. **(A)** HCT116 cells were injected subcutaneously into the dorsolateral quadrant of male nude mice. When the diameter of the subcutaneous tumors reached 100 mm^3^, each animal was treated with the vehicle (Ctrl) or 5-FU (N = 5) for 9 days. **(B)** Relative tumor volume. **(C)** Western blot analysis of GPX4 and FTH1 in tumor tissues from the indicated mice. **(D)** Flow cytometry analysis of the level of ferrous ions in the residual tumors by FeRhoNox-1 staining. **(E)** When the diameter of the subcutaneous tumors reached 100mm^3^, each animal was treated with vehicle, 5-FU alone, RSL3 alone, or a combination of both drugs (N = 5). **(F)** Tumor volumes were measured once every 2 days until 20 days from the initiation of therapy. Tumor volume = (width² × length)/2. **(G)** Images of tumors at the end of the experiment. **(H)** Tumor weight at the end of the experiment. **(I)** Western blot analysis of GPX4 and FTH1 in residual tumors. Data are presented as means ± SD of three independent experiments. Statistical significance was determined using one-way ANOVA with Tukey’s test. *p < 0.05, **p < 0.01, and ***p < 0.001.

The key event in ferroptosis is cell membrane damage associated with peroxidation of lipids (24). GPX4, which is an antioxidant enzyme that reduces ROS, can prevent the formation of toxic lipid peroxides (17, 21). Inhibition of GPX4 activity results in ferroptotic cell death. Given that GPX4 protects cells from chemotherapy-induced oxidative stress (25), we examined whether 5-FU or AZ628 affects *GPX4* expression. We found that the protein and mRNA levels of GPX4 were higher in cells treated with anti-colorectal cancer agents (**Figures 4A–D**, and **Supplementary Figures S3A, B**). In addition, treatment with 5-FU or AZ628 led to an increase in the intracellular levels of ferrous iron, as measured by confocal and flow cytometry of FeRhoNox-1 staining (**Figures 4E, F**, and **Supplementary Figures S3C, D**). Cytoplasmic iron is stored by ferritin heteropolymers, which consist of heavy (FtH) and light (FtL) chains that rise concomitantly in response to the presence of excessive iron (26). We found that the mRNA and protein levels of FTH1 were indeed significantly increased, further confirming the increase in iron upon drug treatment (**Figures 4G–I**, and **Supplementary Figures S3E, F**). Moreover, the levels of GPX4 and FTH1 protein measured by western blotting were upregulated in the xenograft mouse model residual tumors after 5-FU treatment (**Figures 5A–C**, and **Supplementary Figure S4A**). Flow cytometry-based examination of the ferrous iron levels in 5-FU-derived persister tumors revealed increased ferrous iron staining in these residual tumors (**Figure 5D**). These observations suggest that GPX4 expression and ferrous iron levels are upregulated in anti-colorectal cancer drug-tolerant cells and could hence be therapeutic targets for the selective eradication of persister cells. Indeed, RSL3 was specifically lethal to persister cells by downregulating the elevation of GPX4, not FTH1 (**Figures 4J–L** and **Supplementary Figures S3G-I**). These results further confirm GPX4 is so vital for CRC persister cells survival that they are more sensitive to GPX4 inhibition compared to parental cells.

### The Ferroptosis Inducer RSL3 Suppresses CRC Regrowth After 5-FU Therapy Cessation *In Vivo*

To confirm the anti-tumor regrowth effect of GPX4 inhibition after the removal of anti-colorectal cancer agents, we used a subcutaneous tumor mouse model. The xenograft mice were treated with 5-FU alone, RSL3 alone, or a combination of 5-FU and RSL3 (**Figure 5E**). On their own, 5-FU and RSL3 did not affect the body weight of mice in any group (**Supplementary Figure S4B**). Compared with 5-FU alone, the combination treatment (i.e., 5-FU + RSL3) significantly suppressed tumor regrowth after 5-FU cessation (**Figures 5F–H**). RSL3 alone did not reduce tumor volume or weight compared to the control. Consistent with the experiments *in vitro*, RSL3 downregulated the elevated GPX4 in residual tumors, not FTH1 (**Figure 5I**). These results confirm that loss of GPX4 activity inhibited tumor regrowth after the discontinuation of anti-colorectal cancer drug treatment.

### GPX4 Expression Is Upregulated in Colorectal Cancer Patients Undergoing Neoadjuvant Chemoradiotherapy and Correlates With Poor Prognosis for Patients

**Figure 6:**
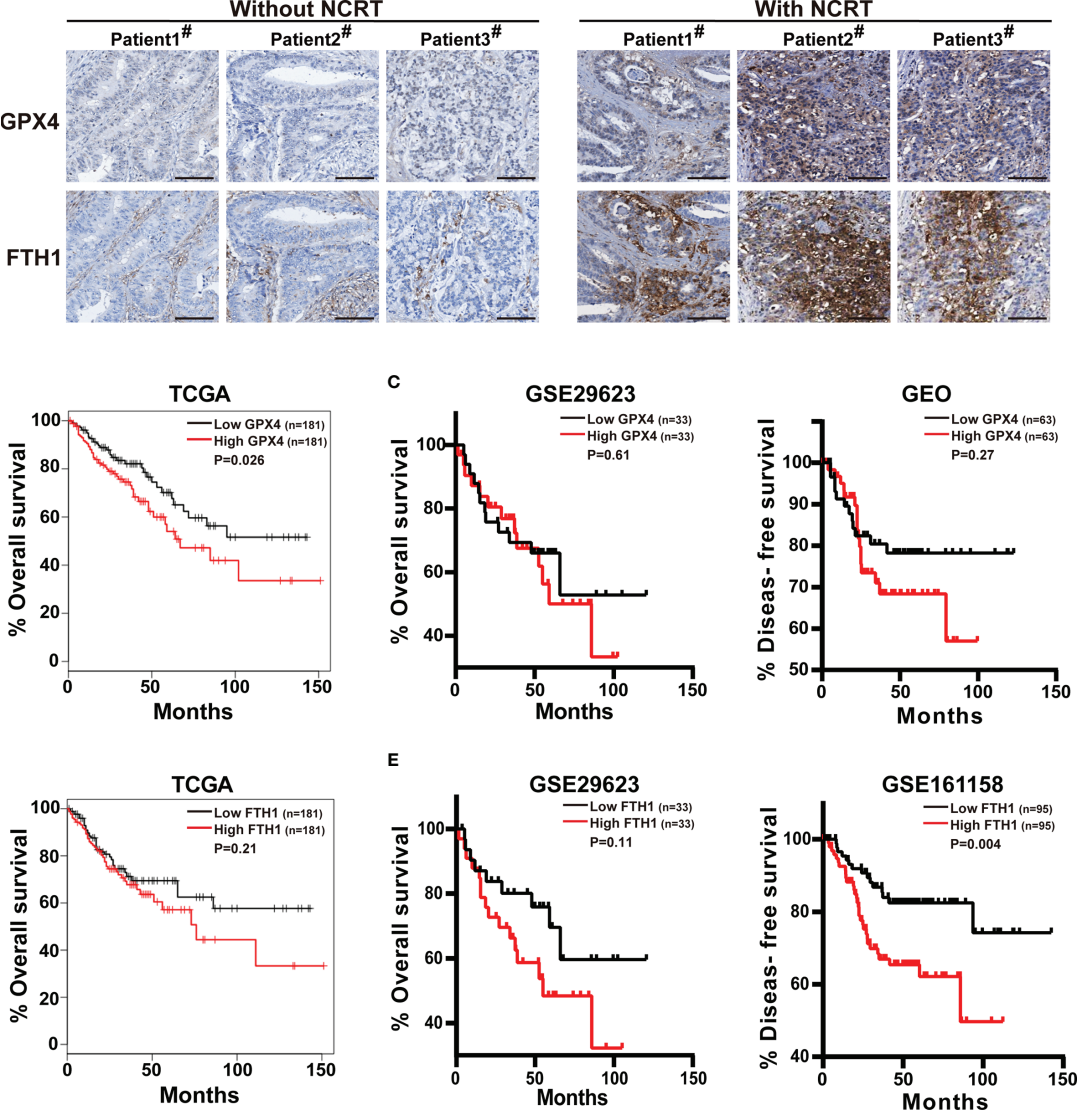
GPX4 expression is upregulated in colorectal cancer patients undergoing neoadjuvant chemoradiotherapy and correlates with poor prognosis for patients. **(A)** GPX4 and FTH1 of biopsies from three colorectal cancer patients with neoadjuvant chemoradiotherapy and three patients without were measured by immunohistochemistry staining (scale: 100 μm). **(B)** Kaplan-Meier curves for overall survival of CRC patients with high vs. low GPX4 expression, according to the TCGA database. **(C)** Kaplan-Meier curves for overall and disease-free survival of CRC patients with high vs. low GPX4 expression, according to the GEO databases. **(D)** Kaplan-Meier curves for overall survival of CRC patients with high vs. low FTH1 expression, according to the TCGA database. **(E)** Kaplan-Meier curves for overall and disease-free survival of CRC patients with high vs. low FTH1 expression levels, according to the GEO databases.

So far, we have demonstrated that GPX4 and FTH1 are upregulated in anti-colorectal cancer drug-tolerant persister cells and tumors. Next, we detected the GPX4 and FTH1 levels in colorectal cancer patients undergoing neoadjuvant chemoradiotherapy by immunohistochemistry, and found both protein levels were up-regulated, compared with the patients without neoadjuvant chemoradiotherapy (**Figure 6A**). Furthermore, we comprehensively analyzed the relationship between expression of GPX4 or FTH1 and survival based on The Cancer Genome Atlas (TCGA) and Gene Expression Omnibus (GEO) databases. TCGA database analysis revealed that patients with high GPX4 mRNA levels had a poorer prognosis than those in the group with low levels (**Figure 6B**). Similarly, GEO database analysis showed a consistent trend, although the difference was not statistically significant (**Figure 6C**). Moreover, patients with high FTH1 mRNA levels had significantly lower overall and disease-free survival than those with low FTH1 mRNA levels (**Figures 6D, E** **)**. These observations suggest that GPX4 and FTH1 are upregulated in patients undergoing neoadjuvant chemoradiotherapy, and high levels of both are associated with recurrence and poor prognosis in colorectal cancer.

## Discussion

Non-genetic mechanisms have recently emerged as important drivers of cancer therapy failure, whereby some cancer cells enter a reversible drug-tolerant persister state in response to treatment (27). Understanding and defining traits of DTP cells is an urgent priority in order to target these cells and prevent the development of drug resistance. Recent studies have reported that the signatures of DTP cells derived from different cancer cell lineages, including ovarian cancer, breast cancer, lung cancer *et al.* (11, 17). However, the characteristics of CRC DTP cells derived from anticancer drugs have not been revealed in detail. In this study, we revealed the traits of CRC DTP cells responses to 5-FU or AZ628, reduced anticancer drug sensitivity, the reversible state, their G0/G1 cell cycle arrest, the upregulation of stemness and mesenchymal markers, and the downregulation of epithelial markers. Recently, it was reported that lung cancer DTP cells exhibit distinct metabolic programs, and enhanced fatty acid oxidation is related to the growth capacity of persister cells, thus highlighting new vulnerabilities that can be targeted (27). Here, we determined the molecular signatures of CRC DTP cells, but the metabolic programs, which have important therapeutic implications, remain elusive and required further study.

We found that the *GPX4* expression and ferrous iron levels were increased in 5-FU- or AZ628-derived tolerant CRC persister cells, which render persister cells sensitive to GPX4 inhibitors-induced ferroptosis. The discovery of 5-FU up-regulation of GPX4 in this study is consistent with the latest reports, and the reproducibility of the results was further confirmed (25). Additionally, GPX4 has also been shown to be upregulated in platinum-tolerant ovarian cancer cells, which sensitizes them to GPX4 inhibitors (17). DTP cells derived from breast cancer exposed to lapatinib have been reported to downregulate a broad range of antioxidant genes, including NRF2 target genes. NADPH, which is a reducing cofactor that has been shown to protect cells from the toxic effects of lipid peroxidation and to prevent ferroptosis, was also decreased in breast cancer persister cells (16). Interestingly, aldehyde dehydrogenase, which is an antioxidant enzyme that protects cells from the potentially toxic effects of ROS, was found to be greatly upregulated in a crizotinib-tolerant gastric carcinoma subpopulation, thereby sensitizing these cells to ALDH inhibitor disulfiram-induced apoptosis (28). The antioxidant enzyme GPX4 uses glutathione as a cofactor to reduce allylic lipid hydroperoxides to their corresponding alcohols (29). Similarly, there may be an impaired antioxidant program in DTP cells that results in compensatory elevation of GPX4, consequently rendering them sensitive to GPX4 inhibitors-induced ferroptosis. However, there is a contradictory variation tendency of GPX4 in drug-tolerant cells. GPX4 has been reported to be specifically reduced in imatinib-derived persister gastrointestinal stromal tumor cells (30). The relationship between drug tolerance and GPX4 levels appears to be complex and may depend on the cancer type or treatment agents, and merits further exploration.

Here, we showed that GPX4 inhibition is a promising strategy to selectively eradicate residual CRC cell reservoirs. The GPX4 inhibitors could downregulate GPX4 and selectively reduce the residual CRC persister cell pool *in vitro*. Moreover, we demonstrated experimentally that co-treatment of xenografts in mice with a 5-FU chemotherapy therapy in conjunction with RSL3 effectively inhibited tumor regrowth. RSL3 is a valuable tool compound for cell culture settings. Although several studies have demonstrated the efficacy of RSL3 in various animal models (20, 31, 32), non-specific effects of RSL3 cannot be excluded given its poor bioavailability *in vivo* (16). Given the therapeutic promise of ferroptosis induction in persister cells, the development of a potent bioavailable GPX4 inhibitor suitable for use with CRC xenografts is an urgent priority.

To date, the majority of studies regarding drug-tolerant persister cells have focused on persister cells from immortalized cell lines, demonstrating that they occur as a small subset of the total tumor population (33). Recently, triple-negative breast cancer-derived xenograft models have also revealed that only a subpopulation of cancer cells can enter the persister state (34). However, CRC DTP cells may have a different pattern of emergence. A pioneering study found that there was no loss of clonal complexity of tumors that entered a drug-tolerant persister state in response to chemotherapy regimens, and the total CRC cell population possessed an equipotent capacity to enter the persister state rather than just a small subpopulation of cells (14). Understanding the mechanisms of DTP induction is critical for the design of more effective therapies that can cure CRC.

In summary, we studied the characteristics of CRC DTP cells derived from the chemotherapy or targeted agents, and the effects of ferroptosis on persister cells. The results showed that CRC DTP cells exhibited reduced sensitivity to the modeling drugs, and were reversible, quiescent, and specifically sensitive to ferroptosis induced by GPX4 inhibitors due to the upregulated GPX4 and ferrous iron.

## Conclusion

In this study, we have identified a ~0.18–7% level of drug-tolerant persister cells with the CRC cell lines response to anticancer drugs, and we determined that there were G0/G1 cell cycle arrest, and upregulation of stemness factors and mesenchymal-like gene expression in the DTP cells. Most importantly, we observed that the levels of GPX4 and ferrous iron of CRC DTP cells were significantly increased, and consequently caused selective sensitivity to GPX4 inhibitor-induced ferroptosis. Our study indicates that ferroptosis of CRC DTP cells presents a novel strategy to prevent chemotherapy or targeted therapy regimen resistance and tumor recurrence.

## Data Availability Statement

The raw data supporting the conclusions of this article will be made available by the authors, without undue reservation.

## Ethics Statement

The studies involving human participants were reviewed and approved by the Institutional Review Board of Peking University Shenzhen Hospital. The patients/participants provided their written informed consent to participate in this study. The animal study was reviewed and approved by the Animal Care and Use Committee of Peking University Shenzhen Hospital.

## Author Contributions

XZ, YM, JM, LY and QS performed the experiments. XZ and GL designed experimental protocols, interpreted data obtained from the experiments, and wrote the manuscript. HW supervised the project, obtained funding and editing the manuscript. GL critically advised and commented for the project, editing the manuscript. All authors contributed to the article and approved the submitted version.

## Funding

This work was supported by funding from the Sanming Project of Medicine in Shenzhen (SZSM201612051), the opening project of State Key Laboratory of Explosion Science and Technology (Beijing Institute of Technology, KFJJ22-16M), National Nature Science Foundation of China (81970467), and CAMS Innovation Fund for Medical Sciences (CIFMS, 2019-I2M-1-003).

## Conflict of Interest

The authors declare that the research was conducted in the absence of any commercial or financial relationships that could be construed as a potential conflict of interest.

## Publisher’s Note

All claims expressed in this article are solely those of the authors and do not necessarily represent those of their affiliated organizations, or those of the publisher, the editors and the reviewers. Any product that may be evaluated in this article, or claim that may be made by its manufacturer, is not guaranteed or endorsed by the publisher.
