# Supplementary material for: Glutathione Peroxidase 4 as a Therapeutic Target for Anti-Colorectal Cancer Drug-Tolerant Persister Cells
Source: Front Oncol. 2022 Jun 3;12:913669. doi: 10.3389/fonc.2022.913669 (PMC9203854; doi:10.3389/fonc.2022.913669)
Supplement: Supplementary file 1 [file DataSheet_1.docx]

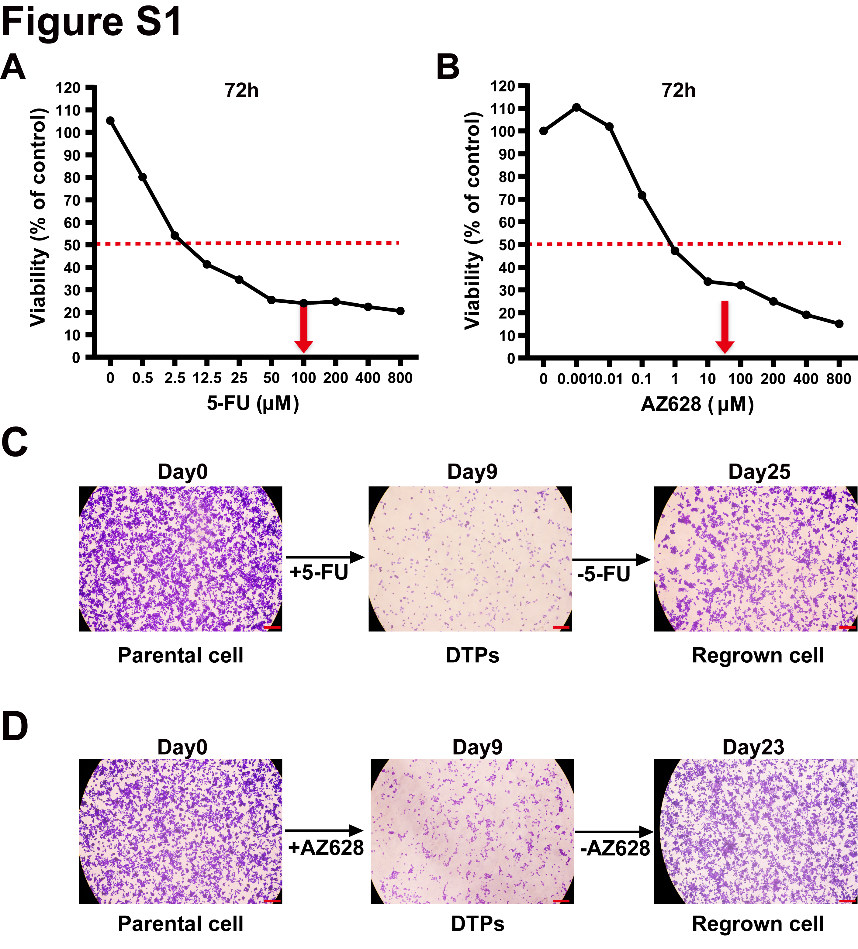


**Figure S1. The drug-tolerant SW620 persister cells model. (A-B)** Survival curves representing the viability of SW620 cells treated with the indicated concentrations of 5-fluorouracil (5-FU) or AZ628 for 72 hours. Each data point is expressed as the percentage of surviving cells relative to untreated controls. The dashed line corresponds to 50% cell killing. The grey arrow indicates the concentration of 5-FU (100 μM) or AZ628 (50 μM) used to generate DTP cells. **(C-D)** Representative pictures of crystal violet staining of SW620 cells that were either untreated (left) or treated with 100 μM 5-FU or 50 μM AZ628 for 9 days (middle, fresh drug was added every 3 days) or removal of drugs (right, scale: 100 μm).


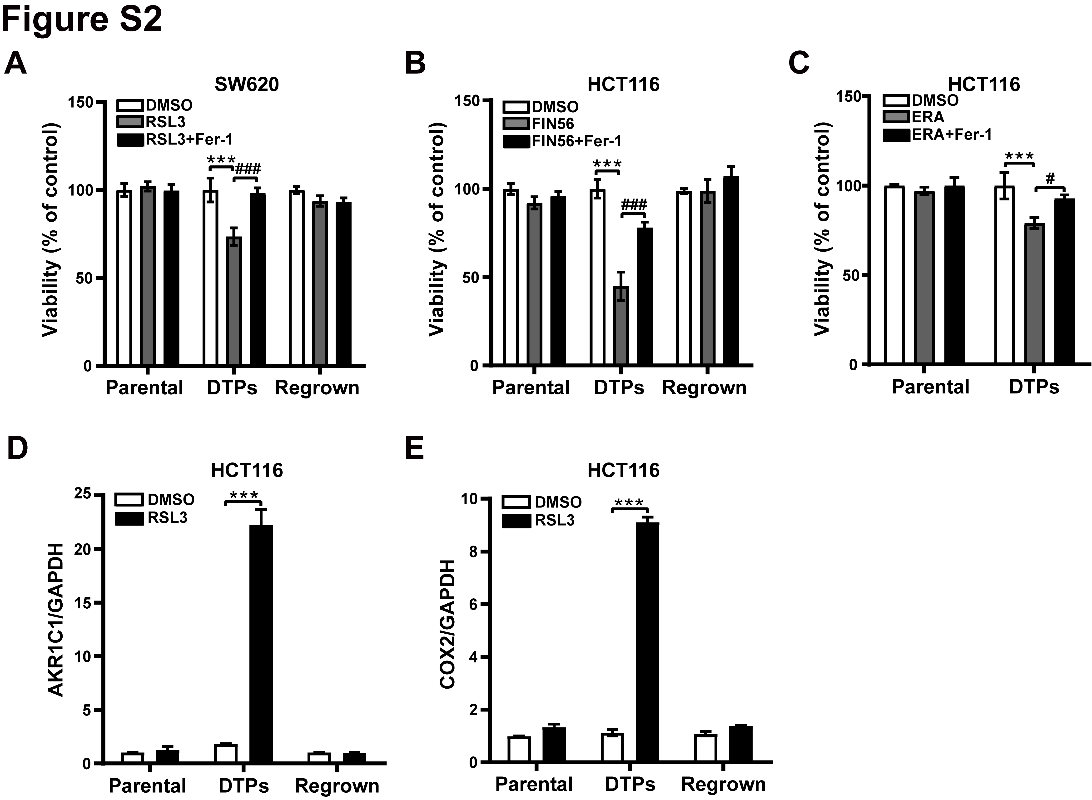


**Figure S2. 5-FU-tolerant CRC persister cells are vulnerable to ferroptosis. (A)** MTT assay of SW620 parental and DTP cells generated by selection in 100 μM 5-FU and regrown for 48 hours ± 2 µM 1S,3R-RSL3 (RSL3) and 2 µM ferrostatin-1 (Fer-1). **(B)** MTT assay of HCT116 parental and 5-FU DTP cells and regrown for 24 hours ± 1 µM FIN56 and 2 µM Fer-1. **(C)** MTT assay of HCT116 parental and 5-FU DTP cells for 24 hours ± 10 µM erastin (ERA) and 2 µM Fer-1. **(D-E)** *AKR1C1* and *COX2* mRNA levels were measured by real-time PCR in the three different conditions under AZ628 treatment with or without 1 µM RSL3 in HCT116. Data are shown as means ± SD of three independent experiments. Statistical significance was determined using 1-way ANOVA with Tukey test. ****p <* 0.001


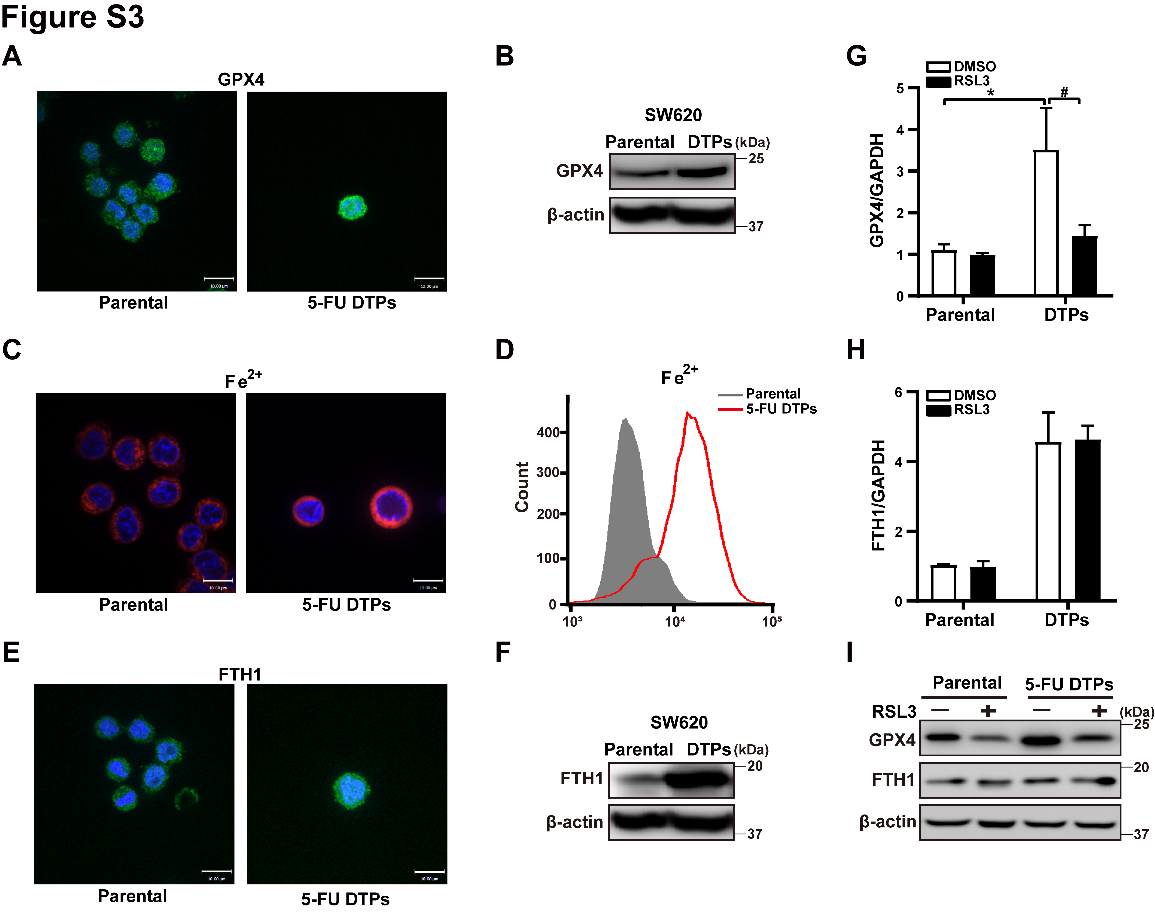


**Figure S3. GPX4 and ferrous iron are upregulated in 5-FU-tolerant SW620 persister cells. (A)** Representative immunofluorescence images of GPX4 staining. Blue and green fluorescence indicate nuclear and GPX4, respectively (scale: 10 μm). **(B)** Western blot analysis of GPX4. **(C-D)** (C) confocal microscopy and (D) flow cytometry analysis of the level of ferrous ions in the cytoplasm using 2 μM FeRhoNox-1 (scale: 10 μm). **(E)** Representative immunofluorescence images of FTH1 staining. Blue and green fluorescence indicate nuclear and FTH1 staining, respectively (scale: 10 μm). **(F)** Western blot analysis of FTH1. **(G-H)** The relative mRNA levels of (G) *GPX4* and (H) *FTH1* measured by real-time PCR in SW620 parental and DTPs derived from 5-FU with or without 2 µM RSL3 for 48 hours. **(I)** Western blot analysis of GPX4 and FTH1. Data are shown as means ± SD of three independent experiments. **p <* 0.05 vs. parental DMSO group; *^#^p <* 0.05 vs. DTPs DMSO group

**
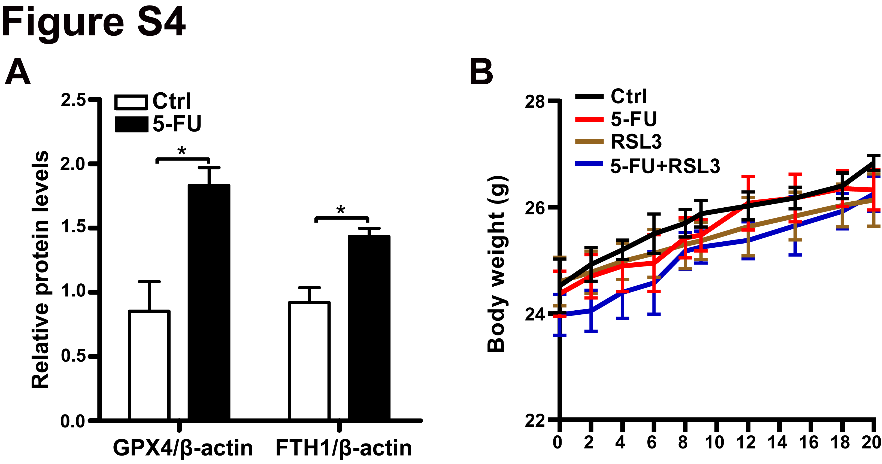
**

**Figure S4. (A)** The GPX4 densitometry relative to β-actin, related to Figure5C. **(B)** Body weights of the mice over time, related to Figure5E. **p* < 0.05

**Supplementary Table S1. All primers used in the study.**

| Genes | Sense | | | Antisense |
| --- | --- | --- | --- | --- |
| Mouse Gapdh | | ACATCAAGAAGGTGGTGAAGC | AGGTGGAAGAGTGGGAGTTG | |
| Mouse Akr1c1 | | TCGTCCAGAACTCGTACGGGT | TCCCGGCTTCATGGCCATTGG | |
| Mouse Cox2 | | TGGGTGTGAAGGGAAATAAGG | CATCATATTTGAGCCTTGGGG | |
| Human GAPDH | | CTCTGCTCCTCCTGTTCGAC | GCGCCCAATACGACCAAATC | |
| Human CD133 | | AGTCGGAAACTGGCAGATAGC | GGTAGTGTTGTACTGGGCCAAT | |
| Human CD44 | | CTGCCGCTTTGCAGGTGTA | CATTGTGGGCAAGGTGCTATT | |
| Human VIM | | GACGCCATCAACACCGAGTT | CTTTGTCGTTGGTTAGCTGGT | |
| Human TWIST | | GTCCGCAGTCTTACGAGGAG | GCTTGAGGGTCTGAATCTTGCT | |
| Human CDH1 | | CGAGAGCTACACGTTCACGG | GGGTGTCGAGGGAAAAATAGG | |
| Human CLDN7 | | AGCTGCAAAATGTACGACTCG | GGAGACCACCATTAGGGCTC | |
| Human TJP3 | | GCTTTGGCATTGCGATCTCTG | GATGTGGTCGCCTGTCTGTAG | |
| Human AKR1C1 | | TCCAGTGTCTGTAAAGCCAGG | CCAGCAGTTTTCTCTGGTTGAA | |
| Human COX2 | | CCCTTCTGGCCTGACACCTTT | TTCTGTACTGCGGGTGGAAC | |
| Human GPX4 | | GAGGCAAGACCGAAGTAAACTAC | CCGAACTGGTTACACGGGAA | |
| Human FTH1 | | CCAGCACCGTTTTTGTGGTT | GCCAATTCGCGGAAGAAGTG | |
